# Roles of Interactions Between Toll-Like Receptors and Their Endogenous Ligands in the Pathogenesis of Systemic Juvenile Idiopathic Arthritis and Adult-Onset Still’s Disease

**DOI:** 10.3389/fimmu.2020.583513

**Published:** 2020-11-05

**Authors:** Ju-Yang Jung, Ji-Won Kim, Chang-Hee Suh, Hyoun-Ah Kim

**Affiliations:** Department of Rheumatology, Ajou University of Medical School, Suwon, South Korea

**Keywords:** damage-associated molecular pattern, toll-like receptor, systemic juvenile idiopathic arthritis, adult onse still disease, inflammation

## Abstract

Systemic juvenile idiopathic arthritis (JIA) and adult-onset Still’s disease (AOSD) are systemic inflammatory disorders that manifest as high-spiking fever, joint pain, evanescent skin rash, and organomegaly. Their pathogenesis is unclear, but inflammation is triggered by activation of the innate immune system with aberrant production of proinflammatory cytokines. Along with extrinsic factors, intrinsic pathways can trigger an unexpected immune response. Damage-associated molecular patterns (DAMPs) induce the activation of innate immune cells, leading to sterile inflammation in systemic JIA and AOSD. These endogenous proteins interact with Toll-like receptors (TLRs), which are pattern recognition receptors, and mediate immune signaling following stimulation by pathogen-associated molecular patterns and DAMPs. Several DAMPs, such as S100 proteins, play a role in the development or severity of systemic JIA and AOSD, in which their interactions with TLRs are altered. Also, the expression levels of genes encoding DAMPs contribute to the susceptibility to systemic JIA and AOSD. Herein, we review reports that TLR and DAMP signaling initiates and/or maintains the inflammatory response in systemic JIA and AOSD, and their correlations with the clinical characteristics of those diseases. In addition, we assess their utility as biomarkers or therapeutics for systemic JIA and AOSD.

## Introduction

Systemic juvenile idiopathic arthritis (JIA) and adult-onset Still’s disease (AOSD) are systemic autoinflammatory diseases characterized by spiking fever, skin rash, polyarticular arthralgia, hepatosplenomegaly, and leukocytosis (1–4). Although the role of the adaptive immune response is limited, activation of the innate immune system plays a pivotal role in both diseases. This has been demonstrated by the activation of innate immune cells and overproduction of proinflammatory cytokines including interleukin (IL)-1β, IL-18, IL-6, and tumor necrosis factor-α (TNF-α) in systemic JIA and AOSD (5, 6). Human leukocyte antigen (HLA) and IL-18, IL-6, and macrophage inhibitory factor (MIF) polymorphisms are associated with the occurrence of systemic JIA and AOSD (7–11). Several viruses, such as rubella, measles, echovirus 7, coxsackievirus, cytomegalovirus, Epstein–Barr virus, parainfluenza, influenza, adenovirus, hepatitis B and C, and parvovirus B19, are reported to trigger both diseases (12–16).

These factors activate the innate immune system; macrophages and neutrophils increase in number, and the levels of markers indicating their activation increase abnormally. Patients with systemic JIA or AOSD had a high serum level of the neutrophil activation marker, CD64 (FcɣRI), the level of which was correlated with disease severity (17, 18). The level of macrophage-colony stimulating factor, which is involved in macrophage differentiation and survival, was increased in the serum of patients with AOSD (19). The serum levels of calprotectin, a calcium-binding protein released during activation of neutrophils and macrophages, and soluble CD163, which is released by activated macrophages, were higher in patients with systemic JIA or AOSD; moreover, their levels were correlated with disease activity (17, 20, 21).

During an innate immune response, pattern-recognition receptors (PRRs) on immune cells interact with pathogen-associated molecular patterns (PAMPs) to induce an immune response. PAMPs include microbial components such as lipopolysaccharides (LPS) from Gram-negative bacteria and viral single-stranded RNA (22). Also, after infectious or non-infectious tissue injury, the release and binding of intracellular or extracellular factors to PRRs on immune cells leads to sterile inflammation. Damage-associated molecular patterns [DAMPs; e.g., high mobility group box 1 (HMGB1), histones, cell-free DNA, and S100 proteins] are potent activators of the immune system.

Several DAMPs play a role in the pathogenesis of systemic JIA and AOSD (23). The level of HMGB1 in serum was elevated in patients with systemic JIA or AOSD, and then downregulated after disease resolution (24, 25). The serum levels of S100A8 (calgranulin A or myeloid-related protein 8, MRP8) and S100A9 (calgranulin B or MRP14) were increased in patients with systemic JIA and AOSD, and these factors were deposited in the skin or lymph nodes of the latter patients (21, 26, 27). Such DAMPs are recognized by members of the PRR family, including Toll-like receptors (TLRs), nucleotide-binding oligomerization domain receptors (NOD-like receptors; NLRs), C-type lectin receptors, and retinoic-acid-inducible gene 1-like receptors, in several inflammatory disorders.

Changes in the adaptive immune system in systemic JIA and AOSD, and the effects of IL-1 and IL-18 on T cell differentiation and activity have been established, suggesting that innate and adaptive immune responses are linked in both disorders (28). The frequencies of circulating Th17 cells were elevated and correlated with disease activity in patients with AOSD, and the proportions of IFN-γ- and IL-17-producing CD4+T cells and IL-17-producing CD3+CD4- T cells were increased in systemic JIA (29, 30). Moreover, an increased population of activated regulatory T cell expressing IL-17 and a prominent Th17 gene expression signature were observed in acute systemic JIA (31). The proportion of CD8+naïve T cells was elevated in AOSD patients and correlated with disease activity (32).

Systemic JIA and AOSD are diagnosed based on clinical manifestations and laboratory results, because there is no reliable biomarker. Although criteria based on combinations of typical clinical and laboratory findings have been established, there is no way to differentiate systemic JIA and AOSD from other conditions (e.g., infections or neoplasms) (33). High levels of inflammatory markers, such as ferritin, have poor diagnostic specificity for systemic JIA and AOSD (34).

Here, we review TLRs and their ligands, and the mechanisms by which they induce an innate inflammatory response. We also summarize the roles of TLRs and their ligands in rheumatic diseases, focusing on systemic JIA and AOSD. An understanding of their pathogenesis will enable the identification and development of reliable biomarkers of both diseases.

## Toll-Like Receptors and Their Ligands

As a link between infection/tissue damage and inflammation, TLRs sense and transfer danger signals to intracellular signaling pathways (35). TLRs interact with different PAMPs and transmit signals *via* specific sets of adaptors and transcription factors in various immune and non-immune cells (**Table 1**). For example, TLR4 recognizes LPS, TLR9 senses unmethylated CpG from bacteria, viruses, or parasites; and TLR7 and 8 recognize viral or parasitic ssRNAs.

**Table 1:** Toll like receptors and their ligands with involved adaptors.

TLR	Localization of immune cell	PAMP	Endogenous ligands
TLR1/2	Cell surface monocyte, macrophage, DC, B cell	Triacyated lipoproteins, peptidoglycan	HSP, HMGB1, β-defensin-3
TLR2	Cell surface monocyte, macrophage, mast cell, B cell	Zymosan, peptidoglycan	HSP, HMGB1, serum amyloid A, surfactant protein A,D, β-defensin-3, antiphospholipid antibody, biglycan
TLR3	Endosomes B cell, T cell, NK cell, DC	dsRNA (viral)	mRNA, tRNA
TLR4	Cell surface/endosomes monocyte, macrophage, DC, mast cell	LPS	HSP, HMGB1, proteoglycans, S100A8, S100A9, S100A12, antiphospholipid antibody, fibronectin, serum amyloid A, oxidized LDL, saturated fatty acids
TLR5	Cell surface monocyte, macrophage, DC,	Flagellin	–
TLR2/6	Cell surface monocyte, macrophage, mast cells, B cell	Diacylated Lipoprotein, Zymosan	–
TLR7	Endosomes monocyte, macrophage, DC, B cell	ssRNA (viral)	ssRNA/IgG complexes, antiphospholipid antibody
TLR8	Endosomes monocyte, macrophage, DC, mast cell	ssRNA (viral)	ssRNA/IgG complexes, antiphospholipid antibody. mircoRNAs
TLR9	Endosomes monocyte, macrophage, DC, B cell, T cell	Unmethylated CpG (viral and bacterial)	Chromatin, IgG complexes, self–DNA including mtDNA

PAMP, pathogen-associated molecular patterns.

TLRs are expressed on macrophages, neutrophils, dendritic cells (DCs), natural killer (NK) cells, mast cells, T- and B-cells, and some types of nonimmune cells (*e*.*g*., epithelial and endothelial cells) (36). Most TLRs are type I transmembrane proteins located in the plasma membrane, intracellular endosomes, or both. TLR2 and TLR4 are extracellular receptors, and TLR3, TLR7, TLR8, and TLR9 are located in the endosomal compartment (37). TLRs comprise extracellular domain-containing leucine-rich repeats (LRRs) and a cytoplasmic Toll/IL-1 receptor (TIR) domain (35, 36). Upon ligand interaction, the homodimerization or heterodimerization of TLRs, with the exception of TLR3, triggers the production of the adaptor molecule myeloid differentiation primary response protein 88 (MYD88), which interacts with IL-1R-activating kinase (IRAK)-4 and IRAK-2. TLR3 and TLR4 interact with TIR domain-containing adaptor molecule 1 (TICAM-1 or TRIF) *via* stimulation by dsRNA viruses (38). The dimerization of IRAK-4 and -2 activates TNF-receptor-associated factor 6 (TRAF6); the final protein complex (Myddosome) induces nuclear transcriptional activity and the transcription of nuclear factor kappa B (NF-κB), interferon regulatory factor (IRF), and AP-1 (39, 40). The various TLR signaling pathways involve different transcription factors, and trigger various cellular responses, resulting in the expression of genes encoding inflammatory cytokines such as type I IFN and IFN-inducible genes. The production of IL-1, IL-6, TNF-α, IL-12, IFNs, chemokines, adhesion molecules, costimulatory molecules, and tissue-degrading enzymes is induced by interactions between ligands and TLRs (41). Diverse factors, ranging from microbial agents to self-DNAs, trigger multiple TLR pathways in a variety of cell types and induce the expression of distinct subsets of genes.

## Roles of TLRs and Their Endogenous Ligands in Sterile Inflammation

TLR-mediated stimulation is implicated in diverse diseases, including infections, sepsis, autoimmune diseases, and malignancies. DAMPs interact with TLRs as endogenous ligands and initiate signal transduction, inducing inflammatory responses. Most DAMPs, which include nucleic acids, intracellular proteins, and extracellular matrix components, are released by damaged tissues or dying cells. Their interactions are implicated in the pathogenesis of sterile inflammatory conditions, including rheumatic diseases, cancer, and wound healing.

S100A8 and S100A9 are released during the apoptosis or necrosis of neutrophils and monocytes or the formation of neutrophil extracellular traps (NETs) (42). They interact with TLR4 and receptor for advanced glycation end products (RAGE), and S100A8/S100A9-TLR4 signaling in human monocytes has effects similar to LPS-TLR4 signaling. HMGB1 is a proinflammatory mediator that binds several PRRs, including RAGE, TLR4, TLR9, C-X-C chemokine receptor type 4 (CXCR4), and T cell immunoglobulin mucin-3 (TIM-3) (43). HMGB1 is released during apoptosis or necrosis, acting as a DAMP, and inflammatory cytokines including TNF-α and IFN-ɣ enhance the release of HMGB1 (44, 45). Extracellular HMGB1 binds to TLR4 adaptor myeloid differentiation factor-2 (MD-2), triggering the activation of NF-κB and the transcription of proinflammatory cytokines (46). In addition, the HMGB1–CpG-ODN complex binds to TLR9, promoting cytokine production (37). As an acute-phase reactant and DAMP, serum amyloid A (SAA) can induce proinflammatory cytokines during injury, sterile inflammation, and infection, and SAA promotes the production of IL-1β *via* activation of the NLRP3 inflammasome (47). The action of SAA is dependent on TLR signaling. SAA activity decreases in TLR4 deficiency and SAA induces G-CSF and IL-8/CXCL8 *via* TLR2 (48–50).

TLR3, TLR7, and TLR9 on endosomes are activated by self-nucleic acids such as self-DNA and RNA-protein particles (41). Single-stranded RNA, double-stranded RNA, and unmethylated CpG DNA stimulate the TLR7, TLR3, and TLR9 signaling pathways, respectively. Internalization of self-nucleic acids activates the TLR7 and TLR9 signaling pathways and stimulates transcription of IFN-α in patients with autoimmune diseases, such as SLE.

TLR signaling promotes activation of the NLR family pyrin domain-containing 3 (NLRP3) inflammasome, which is a cytoplasmic protein complex that modulates the innate immune response (51). TLR signaling *via* MyD88 or TRIF stimulates transcription of *Nlrp3* and synthesis of pro-IL-1β, resulting in inflammasome assembly and activity (52). TLR signaling and inflammasome activity also promote pyroptosis and inflammatory caspase-dependent lytic cell death, inducing the release of IL-1β, IL-18, and HMGB1 in sterile inflammation (53, 54).

## Roles of TLRs and Their Ligands in Rheumatic Diseases

Rheumatoid arthritis (RA), which is the most common autoimmune disease, is characterized by joint inflammation and destruction. A variety of genetic and environmental factors are related to the chronic inflammatory response in RA. TLR signaling is implicated in the development and maintenance of RA, and potential strategies targeting TLR signaling are currently under investigation (55). TLR2, TLR3, TLR4, and TLR7 were highly expressed on synovial tissue, and synovial tissue macrophages, and the levels of TLR2 and TLR4 expression were associated with IL-12 and IL-18 levels in synovial tissue in RA (56, 57). Endogenous DAMPs, such as heat shock proteins, fibronectin, HMGB1, and S100 proteins, activate synovial macrophages or DCs *via* TLR signaling pathways (58, 59). TLR2 signaling induces the release of chemokines by synovial fibroblasts, and the MYD88-dependent pathway is involved in joint inflammation in RA (60). Synthetic dsRNA and necrotic synovial fluid stimulated TLR3 on synovial fibroblasts, inducing the production of IFN-β, CXCL10, CCL5, and IL-6. Also, TLR3 and TLR4 signaling in synovial fluid macrophages resulted in overproduction of TNF-α and IL-6 (61, 62). TLR-3 and TLR-7 were highly expressed in the RA synovium, and TLR2 and TLR4 signaling in DCs from patients with RA triggered the production of inflammatory mediators (63). TLR7 expression in RA monocytes induced TNF-α production and correlated with the disease activity of RA (64).

TLR2 signaling promotes joint inflammation during the acute phase, and TLR4 promotes matrix metalloproteinase (MMP)-mediated cartilage destruction, osteoclast formation, and IL-17 production in the chronic phase (65). A study using a serotonin receptor antagonist showed that TLR8 signaling induced TNF production in RA (66). The expression of TLR3 and TLR7 on DCs and synovial fibroblasts was controlled by type I IFN, but not by IL-1β, IL-18, or TNF-α (67). TLR5 is strongly expressed in the synovium of RA patients, and ligation of TLR5 induces TNF-α and promotes monocyte migration to synovial tissue and osteoclastic development of myeloid cells (68). Some TLR4, TLR5, TLR7, and TLR9 polymorphisms are associated with susceptibility to RA (69). TLR4 polymorphisms are also associated with shared epitope and disease activity (70).

Systemic lupus erythematosus (SLE) is a systemic autoimmune disease involving multiple organs, an autoantibody response, and immune complex deposition. The TLR7/MyD88 signaling pathway in plasmacytoid DCs and B cells plays a role in the pathogenesis of SLE (71). After internalization, immune complexes bound to DNA or RNA interact with endosomal TLR7 and TLR8, promoting a type I IFN response (72). TLR7 was overexpressed in models of severe lupus, and in patients with SLE, and the inhibition or attenuation of TLR7 signaling ameliorated the inflammatory response in the lung and kidney of lupus-prone mice (73, 74). TLR9 expression induced the generation of autoantibodies (*e*.g., anti-dsDNA antibodies) and B-cell activation *via* immune complexes (75). Although TLR9 signaling contributes to the development of lupus nephritis, enhanced TLR9 signaling prevents severe manifestations and defective TLR9 expression promotes inflammation in murine lupus (76–78). The role of TLR9 in SLE is controversial. TLR8 signaling modulates TLR7 activation, and deletion of TLR8 promotes autoantibody production and inflammation (79).

In addition, blockade of TLR signaling in major immune cells attenuates the inflammatory response in SLE, confirming a role for TLR in its pathogenesis. The inhibition of IRAK-4, which involves the TLR7 signaling pathway, on plasmacytoid DCs not only reduces the expression of IFN-responsive genes but also ameliorates inflammation in lupus nephritis (80). A TLR inhibitor prevented tissue damage caused by immune complex deposition and cellular infiltration, and alleviated collagen-induced arthritis and the manifestations of SLE (81). Mutations in TLR-trafficking chaperone, which suppresses TLR7 signaling, ameliorated systemic inflammation (82). TLR7 and TLR9 polymorphisms are associated with SLE susceptibility, particularly in Asians (83).

Sjögren syndrome is an autoimmune disease characterized by lymphocytic infiltration and inflammation of the salivary and lacrimal glands. There is aberrant expression of TLRs on PBMCs, minor salivary gland (MSG) biopsy tissue and salivary gland epithelial cells (SGECs) in Sjögren syndrome. TLR2, TLR3, and TLR4 were strongly expressed on the SGECs of patients with Sjögren syndrome, and TLR2 signaling induces the production of IL-23/IL-17 *via* IL-6 and signal transducer and activator of transcription (STAT) in the NF-κB pathway in Sjögren syndrome (84, 85). TLR7, TL9, and TLR7/8 signaling is also associated with a salivary inflammatory response involving antigen presentation and the secretion of proinflammatory cytokines (86).

Systemic sclerosis (SSc) is an autoimmune disease characterized by fibrosis of the skin and/or internal organs and vasculopathy (87). Autoantibody-producing or autoreactive cells induce endothelial activation and progressive fibrosis in SSc. TLR signaling pathways play roles in the pathogenesis of SSc, and their pharmacological inhibition ameliorates disease progression. The level of fibronectin, an endogenous TLR4 ligand, is increased in the serum and skin tissue of patients with SSc, and disruption of TLR4 signaling abrogated collagen production and myofibroblast differentiation (88). Also, the level of the extracellular matrix glycoprotein tenascin-C is elevated in the serum, fibroblasts, and skin lesions of patients with SSc, inducing collagen gene expression and myofibroblast transformation *via* TLR4 signaling (89). Mitochondrial DNA and CpG oligonucleotides trigger TLR9 signaling, leading to TGFβ production and fibroblast activation in patients with SSc (90). Liquid crystalline complexes composed of CXCL4 and self-DNA or microbial DNA amplify the activation of plasmacytoid DCs and IFN-α production *via* TLR9 signaling in SSc (91).

Antiphospholipid antibody syndrome is characterized by recurrent thrombosis and complications of pregnancy associated with an autoimmune-mediated inflammatory response. Antiphospholipid antibodies (aPLs) include anti-cardiolipin antibodies (aCLs), lupus anticoagulant (LAC), and anti-β2 glycoprotein I (GPI) antibodies. aPLs induce an inflammatory response by interacting with TLR2, TLR4, TLR7, TLR8, and TLR9 (92–95). In placental inflammation or thrombosis, anti-β2 GPI activates TLR4, impairing autophagy and activating the inflammasome in endothelial cells (96, 97).

Gout, which is the most common rheumatic disease, is characterized by an acute inflammatory response against monosodium urate monohydrate (MSU) crystals in the joints. TLR2 and TLR4 signaling *via* MyD88 is triggered by MSU crystals, and induces the production of proinflammatory cytokines (including IL-1β and TNF-α) by activating the NALP3 inflammasome (98–100). S100A8 and S100A9 were released by MSU crystal-activated phagocytes, and stimulated IL-1β secretion in a TLR4-dependent manner. The S100A8 and S100/A9 levels were elevated in patients with gout and were correlated with disease activity (101). An inhibitor of the NALP3 inflammasome and TLR2 suppressed MSU crystal uptake by macrophages, and alleviated swelling and pain in MSU-injected joints (102).

**Table 2** summarizes the putative pathological roles of TLR and its ligands in several rheumatic diseases.

**Table 2:** Toll like receptors in rheumatic diseases.

Disease	Involved TLRs	Expressed cells	DAMP			Function	References
Rheumatoid arthritis	TLR2	Synovial fibroblast synovial tissue, DC	HSP60, HSP70, gp96, HMGB1, biglycan, serum amyloid A			Production of IL-1β and TNF-α Secretion of GCP-2, RANTES, MCP-2	(56–58, 60)
	TLR3	Synovial fibroblasts	dsRNA			Induction of IL-6, MMP-3, MMP-13, IFN-β, CXCL10, CCL5	(61–63)
	TLR4	Synovial fibroblast, synovial tissue, macrophage, DC	HSP22, HSP60, HSP70, EDA fibronectin, fibrinogen, low molecular weight hyaluronic acid, HMGB-1, biglycan, S100A8			Production of TNF-α and IL-6 Development of MMP-mediated cartilage damage and osteoclast formation Induction of cell efflux, chondrocyte death, proteoglycan depletion, cartilage destruction, bone erosion	(58–60, 63, 65)
	TLR5	Synovial macrophage, PBMC	Flagellin			Production of TNF-α Monocyte infiltration and osteoclast maturation	(64)
	TLR7	Synovial fibroblast, DC, macrophage	ssRNA/IgG complex			Induction of TNF-α	(64)
	TLR8	Synovial membrane cultures,	ssRNA/IgG complex			Induction of TNF	(66)
Systemic lupus erythematosus	TLR4	Plasma cell, macrophage, monocyte, renal tissue	Anti-dsDNA antibody,			Autoantibody production Development of glomerulonephritis Activation of NLRP3 inflammasome Secretion of IL-1β Induction of TNF-α, IL-6, IL-23, IL-10	(76, 81)
	TLR7	B cell, plasmacytoid DC		snRNA-Ag complex		Release of IFN-α	(71–74, 80, 82)
	TLR9	B cell, spleen monocytes		snRNA-Ag complex		Induction of type I IFN and TNF-α Regulation of autoantibody production via TLR7	(75–79)
Sjögren syndrome	TLR2	Minor salivary gland, salivary gland epithelial cells, PBMC			Biglycan, decorin	Production of IL-17 and IL-23	(84, 85)
	TLR4	Minor salivary gland, salivary gland epithelial cells, PBMC. Splenocyte			Biglycan, decorin	Production of IL-17 and IL-23 Production of salivary IL-6, MCP-1, TNF-α	(84)
	TLR7	PBMC, plasmacytoid DC			–	Induction of IFN	(86)
	TLR9	Minor salivary gland, salivary gland epithelial cells, PBMC, B cell			–	Release of IL ‐8, IL-15, MCP ‐1, IL-6, IL ‐2R	(86)
Systemic sclerosis	TLR4	Fibroblast, skin tissue			Fibronectin, tenascin-C	Collagen production Fibroblast differentiation	(88, 89)
	TLR9	Fibroblast, skin tissue			Mitochondrial DNA and CpG oligonucleotide immune complexes composed of CXCL4 and microbial DNA	TGF-β production and fibroblast activation Activation of plasmacytoid DC IFN-α production	(90, 91),
Antiphospholipid antibody syndrome	TLR2	Embryonic fibroblast, monocyte			anti-beta2-glycoprotein 1 IgG	Induction of MCP-1, ICAM-1, IL-6 Inhibition of endothelium-dependent relaxation Induction of tissue factor expression	(93)
	TLR4	Endothelial cells, trophoblast, macrophage, monocyte			anti- beta2 glycoprotein 1, anti-cardiolipin	Activation of inflammasome Secretion of IL-8, MCP-1, GRO-α, and IL-1β Inhibition of endothelium-dependent relaxation Induction of tissue factor expression	(92, 96, 97)
	TLR7	Monocytes, plasmacytoid DC			ssRNA	Induction of IL-1β and caspase-1	(95)
	TLR8	PBMC, Monocytes, plasmacytoid DC			IgG from the patient with APS	Secretion of TNF-α	(94, 95)
Gout	TLR2	PBMC, Macrophage, Chondrocyte			MSU crystals	Activation of inflammasome Induction of IL-1β and TNF-α	(98, 99, 102)
	TLR4	PBMC, Macrophage			MSU crystals, S100A8 and S100A9	Stimulation of IL-1β secretion	(98, 100, 101),

DAMP, damage-associated molecular pattern; DC, dendritic cell; PBMC, peripheral blood mononuclear cell; TLR, toll like receptor; IL, interleukin; TNF, tumor necrosis factor; GCP, granulocyte chemotactic protein; MCP, monocyte chemoattractant protein; RANKLE, receptor activator of nuclear factor kappa-B ligand; HSP22, heat shock protein B8; MMP, matrix metalloproteinase; ICAM, intercellular adhesion molecule; CXCL, CXC motif chemokine; CCL, Chemokine C-C motif ligand; TGF, transforming growth factor; MSU, monosodium urate.

## Roles of TLR and Their Ligands in the Pathogenesis of Systemic JIA and AOSD

**Figure 1:**
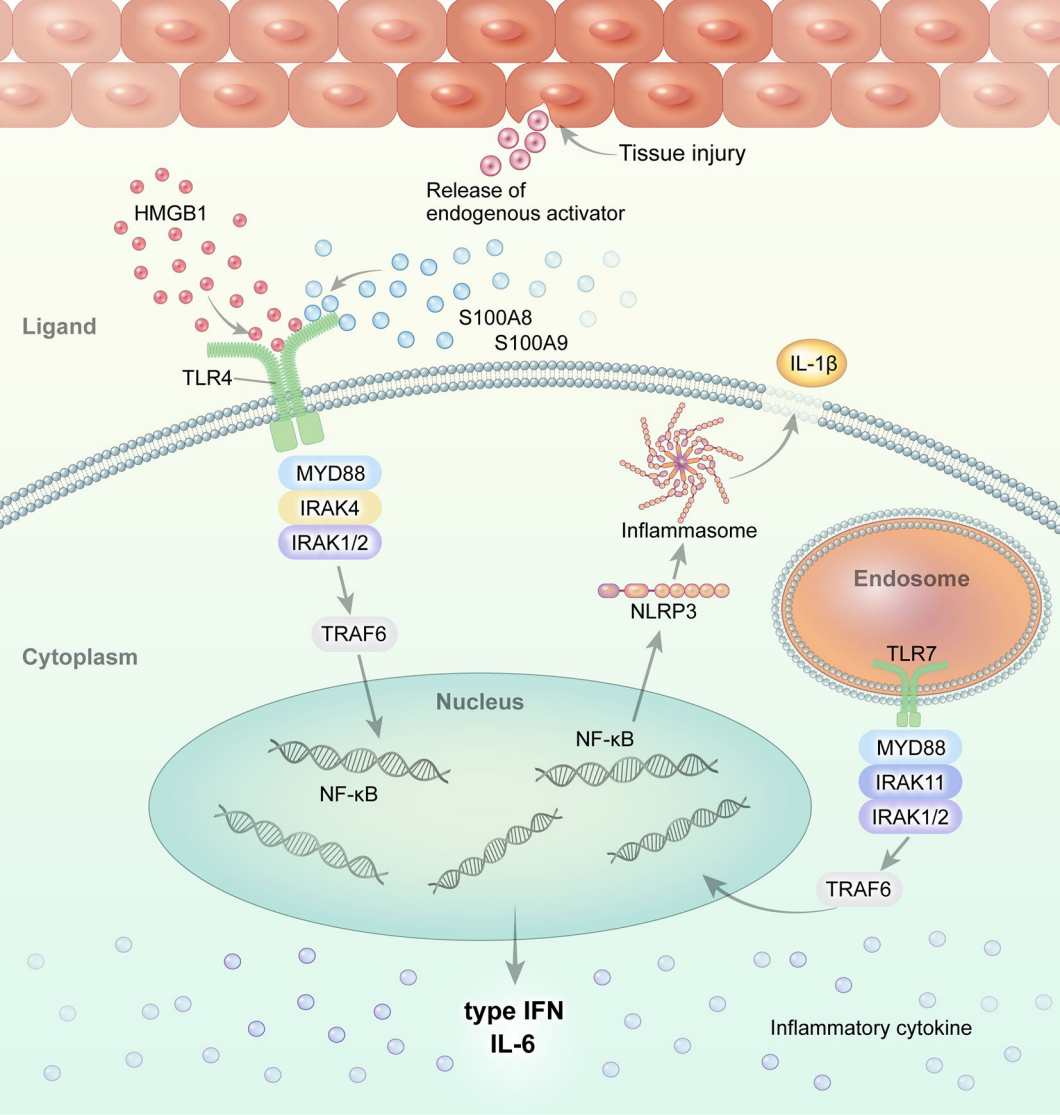
Overview of TLR signaling and the endogenous DAMP pathway in systemic JIA and AOSD. Regarding the role of TLR and its ligands in the pathogenesis of systemic JIA and AOSD, current evidence suggests that endogenous ligands, such as S100A8, S100A9, and S100A8/A9, and HMGB1, interact with and stimulate the TLR4 pathway. Activated TLR4 and TLR7 induce NLRP3 inflammasome activation and the secretion of IL-1β in systemic JIA and AOSD.

Macrophages and monocytes are highly activated in systemic JIA and AOSD. Altered TLR signaling and its ligands have been implicated in the pathogenesis of systemic JIA and AOSD (**Figure 1**). We review the role of TLR and its DAMP molecules in the pathogenesis of systemic JIA and AOSD.

### Interaction of S100 Proteins and TLR4

S100A8, S100A9, S100A8/A9 (calprotectin), and S100A12 are calcium-binding proteins released from activated phagocytic myeloid cells that act as proinflammatory endogenous TLR4 ligands during sterile inflammation (42). The interaction of S100 proteins and TLR4 in the context of sterile inflammation or tissue injury is independent of the presence of PAMP. S100A8, S100A9, and S100A12 induce proinflammatory cytokines *via* TLR4 (103–106). S100A8 interacts with the TLR4/MD2 complex, and S100A8/S100A9 activities are locally restricted through hiding the TLR4/MD2-binding site by (S100A8/S100A9)_2_ tetramer formation (107).

S100A8/S100A9 binding to TLR4 also induces the transcription of inflammatory cytokine genes, such as IFN regulatory factor 3 (IRF3) (108). Injection of S100A8 enhanced the expression of the Fcɣ receptor (FcɣR) on macrophages in the synovium of a chronic experimental arthritis mice model, while up-regulated expression of FcɣR was abrogated in the synovium of TLR4 knockout mice (109). In an inflammatory autoimmune disease model, S100A8 and S100A9 expression was up-regulated and contributed to the development of IL-17-expressing CD8+ T cells (110). The interaction of S100A8/A9 and TLR4 upregulates IL-17 expression in CD8+ T cells.

Serum S100A8 and S100A9 levels were highly elevated in patients with systemic JIA or AOSD (26, 65). Serum levels of S100A8/A9 were significantly higher in patients with systemic JIA compared to those with systemic infection or other types of JIA, and were correlated with IL-1β expression on phagocytes (111). Serum S100A8/A9 levels were elevated in patients with AOSD, including those with lymphadenopathy and skin rash (21, 26, 112). The serum S100A12 levels were also increased in systemic JIA and AOSD, suggesting pathological roles as DAMPs (113, 114). As endogenous ligands, S100A8/A9 and S100A12 activate TLR4 and RAGE signaling, promoting the production of proinflammatory cytokines such as IL-1β, IL-6, and TNF-α (103, 115, 116). Elevated levels of these cytokines activate inflammatory cells, including neutrophils and monocytes, to produce the TLR4 ligand S100, leading to disease progression. Furthermore, a recent study identified neutrophil activation in both active and clinically inactive systemic JIA patients, characterized by the expression of proinflammatory genes, such as *S100A8*, and inflammasome components, reflecting persistent innate immune activation (117). They showed that neutrophils in patients with both active disease and longstanding clinically inactive disease had significantly increased capacity to release S100A8/A9 upon activation. However, there was no association between two functional single nucleotide polymorphisms in TLR4 and susceptibility to JIA (118). Furthermore, TLR4 expression is reportedly significantly decreased on the monocytes of patients with SLE and JIA, including systemic JIA. Reduced TLR4 expression was suggested to be a cause of chronic arthritis or the result of a feedback loop (119).

### Interaction of HMGB1 and TLR4

HMGB1, which is a DNA-binding protein released by necrotic or damaged cells, is an extracellular DAMP that links tissue injury to the innate immune response (43). HMGB1 binds to RAGE and TLR4, propagating inflammatory signals. HMGB1 is implicated in systemic inflammation in sepsis, liver injury, arthritis, and SLE. The serum HMGB1 level is elevated in patients with AOSD and systemic JIA compared to healthy controls (HCs) (24, 25), and is correlated with the systemic AOSD score (24). HMGB1 is released from NETs, which are important in the pathophysiology of AOSD (120). The serum levels of NET molecules (including cell-free DNA, myeloperoxidase (MPO)-DNA complex, and α-defensin) were increased in patients with AOSD (121). The serum from active AOSD patients induced NETosis in neutrophils from HCs. NET molecules induced IL-1β production by monocytes, representing a novel pathogenic mechanism of AOSD. Therefore, NET molecules, as ligands of TLR4, might be associated with activation of the TLR signaling pathway in systemic JIA and AOSD.

### NLRP3 Inflammasome and TLR7

TLR7 signaling, which is triggered by nucleic acids from damaged host cells, contributes to chronic inflammatory disorders. The expression of TLR7 on circulating precursors of myeloid DCs (pre-mDCs) and mDCs was markedly elevated in patients with AOSD compared to HCs (51). The transcript levels of TLR7, Myd88, IRAK4, TRAF6, and IFN-α were correlated with the serum IL-1β and IFN-α levels in patients with AOSD. After remission, the expression of TLR7 on circulating pre-mDCs, and the transcript levels of TRAF6 and IRAK4, were significantly decreased. NLRP3 inflammasome activation *via* the TLR7-MyD88 pathway promotes secretion of IL-1β and IL-18, leading to chronic inflammation. The levels of the NLRP3 inflammasome and its byproducts were significantly elevated in patients with AOSD and correlated with disease activity (122). A TLR7 agonist upregulated the levels of NLRP3 inflammasome pathway components (caspase-1, IL-1β, and IL-18) in patients with AOSD, but not in HCs.

### IL-6

IL-6, as one of the proinflammatory cytokines upregulated in systemic JIA and AOSD, plays an important role in systemic inflammation. IL-6 enhances the TLR-induced inflammatory response *in vivo* and *in vitro* (123). The administration of IL-6 increased the levels of IL-1β, and some chemokines, in peripheral blood monocytes and synovial fluid mononuclear cells (RA synoviocytes), suggesting amplification of the TLR signaling-mediated cytokine and chemokine response (124). Therefore, an elevated IL-6 level may upregulate TLR signaling in systemic JIA and AOSD.

### Serum Amyloid A and TLRs

SAA is synthesized in response to inflammatory stimuli, and induces IL-β production by activating the NLRP3 inflammasome; this is involved in the pathogenesis of systemic JIA and AOSD. Circulating SAA levels were higher in JIA patients, and correlated with the disease activity of JIA (125). A SAA gene polymorphism study revealed that one T allele of rs12218 is associated with disease susceptibility in AOSD patients (126). SAA plays a role in sterile inflammation through activation of TLR2 or TLR4 and their signal pathways. The activation of TLR2 or TLR4 signaling by SAA might contribute to the inflammation seen in systemic JIA and AOSD, although further research on this is needed.

## TLR and Their Ligands as Biomarkers of Systemic JIA and AOSD

Few studies have evaluated the role of TLRs as biomarkers of systemic JIA and AOSD. Moreover, only a few studies have evaluated the utility of serum levels of their ligands as biomarkers, because cell surface TLR levels are difficult to evaluate. TLR4 ligands, such as S100A8/A9, S100A12, HMGB1, and NET molecules, have become reliable biomarkers for diagnosing and evaluating disease activity in systemic JIA and AOSD. The serum level of the TLR4 ligand S100A8/A9 was correlated with disease activity (based on acute-phase reactants and subjective assessments), and an elevated level during clinical remission was predictive of further disease flares in systemic JIA patients (20). S100A8/A9 was superior to C-reactive protein (CRP) for differentiating systemic JIA from other autoinflammatory syndromes and systemic undifferentiated recurring fever syndrome (127). The serum S100A8/A9 level was correlated with the drug response, suggesting its utility for monitoring disease activity in subclinical systemic JIA (20). Similarly, the serum levels of S100A8 and S100A9 were elevated in patients with AOSD, and were correlated with markers of disease activity, including the systemic disease score (21). Furthermore, the sensitivity and specificity of S100A8/A9 for differentiation of AOSD were 63 and 80.1%, respectively (128). Data on the role of S100A12 as a biomarker are similar to those on S100A9/A9 in systemic JIA and AOSD patients. Serum S100A12 levels were elevated in active AOSD patients relative to HCs and correlated with systemic inflammatory markers, such as ESR, CRP, and ferritin (114). The S100A12 levels were also elevated in patients with systemic JIA compared to patients with infection and HCs (113). The sensitivity and specificity of S100A12 for distinguishing between infection and systemic JIA were 66 and 94%, respectively. One study investigated whether patients with systemic JIA at risk of relapse could be identified using biomarkers, and found that the best single biomarker for predicting flare was S100A12 (129). These data suggest that serum S100A8/A9 and S100A12 levels are good biomarkers for diagnosing systemic JIA and AOSD, predicting relapse, and evaluating disease activity. The utility of serum HMGB1 as a biomarker of systemic JIA and AOSD is limited. The HMGB1 level was higher in patients with AOSD than in HC, but was weakly correlated with the CRP level and systemic score (24). The HMGB1 level was also higher in patients with systemic JIA than in HC, and was associated with serositis and hepatosplenomegaly (25). Another study found positive correlations of the serum level of HMGB1 with ESR, CRP, and α2 globulin in patients with JIA, including systemic JIA. In a NET study, patients with AOSD had higher levels of cell-free DNA and NET-DNA complexes, and their neutrophils released more NETs, compared to HCs (130). The serum levels of cell-free DNA, MPO-DNA, and α-defensin were significantly increased in patients with AOSD compared to HCs (121). Furthermore, these levels were correlated with the levels of several disease-activity markers, and neutrophil elastase and MPO-positive inflammatory cells were detected in the lymph nodes and skin of patients with active AOSD.

One study evaluated the frequencies of TLR7-expressing pre-mDCs and mDCs by flow cytometry in patients with AOSD and SLE, as well as in HC (51). The levels of TLR7 signaling molecules were elevated and positively correlated with disease activity in patients with AOSD.

## Conclusion and Future Perspectives

We have reviewed the roles of TLRs and their ligands, as PRRs and DAMPs, in aggravating inflammation, including sterile inflammation, in two rheumatic diseases (systemic JIA and AOSD). TLRs and their ligands contribute to inflammation in patients with systemic JIA and AOSD. The levels of DAMPs, such as S100 proteins, HMGB1, and MPO-DNA complex, are elevated in the blood of patients with active AOSD or systemic JIA, suggesting that they aggravate inflammation by activating TLR4 in inflammatory cells. Furthermore, the TLR4 ligands S100A8/A9 and MPO-DNA complex have potential as biomarkers for diagnosis and disease activity in patients with systemic JIA and AOSD.

Although the triggers of inflammation are unknown, viral or bacterial infection, as well as environmental factors, could act as danger signals promoting TLR activation. This could in turn lead to the activation of neutrophils and macrophages, and to the production of proinflammatory cytokines such as IL-6 and DAMPs, *via* activated neutrophils and macrophages. This would sustain sterile inflammation in systemic JIA and AOSD. However, some questions remain to be resolved. The first concerns whether TLR signaling is triggered, or occurs due to the loss of inhibitory signals, during initial inflammation. The second concerns which subset of patients is more likely to develop systemic JIA or AOSD even after being exposed to similar environmental factors or viral/bacterial infections. Therefore, further studies should determine the genetic factors associated with onset or exacerbation of the initial TLR responses. The third question concerns whether the TLR–TLR ligand response is associated with chronic features of systemic JIA and AOSD; this is currently unclear, despite the known link between initial acute inflammation and TLR responses. Further basic and clinical research, including large, multicenter, prospective studies, is needed to confirm the role of TLRs and their ligands in systemic JIA and AOSD. The current findings enhance our understanding of the pathogenesis of systemic JIA and AOSD, and will facilitate the development of diagnostic and prognostic biomarkers, as well as novel therapeutics targeting TLR signaling in systemic JIA and AOSD.

## Author Contributions

All authors listed have made a substantial, direct and, intellectual contribution to the work and approved it for publication.

## Funding

This work was supported by grants from the Basic Science Research Program through the National Research Foundation of Korea (NRF) funded by the Ministry of Education, Science and Technology [Grand Number. 2019R1A2C1005988].

## Conflict of Interest

The authors declare that the research was conducted in the absence of any commercial or financial relationships that could be construed as a potential conflict of interest.
